# Supplementary material for: ELAVL1a is an immunocompetent protein that protects zebrafish embryos from bacterial infection
Source: Commun Biol. 2021 Feb 26;4:251. doi: 10.1038/s42003-021-01777-z (PMC7910469; doi:10.1038/s42003-021-01777-z)
Supplement: Supplementary file 2 — Supplementary Information [file 42003_2021_1777_MOESM2_ESM.pdf]

ELAVL1a is an immunocompetent protein that protects zebrafish embryos from bacterial infection

Shousheng Ni[1], Yang Zhou[1], Lili Song[1], Yan Chen[1], Xia Wang[1], Xiaoyuan Du[1], Shicui Zhang[1,2*]

[1]Institute of Evolution & Marine Biodiversity and Department of Marine Biology, Ocean University of China, Qingdao 266003, China

[2]Laboratory for Marine Biology and Biotechnology, Pilot National Laboratory for Marine Science and Technology (Qingdao), Qingdao 266003, China

***Corresponding author：**

Shicui Zhang

Department of Marine Biology

Ocean University of China

5 Yushan Road, Qingdao, China

Phone: +86 532 82032787

Fax: +86 532 82032787

E-mail: sczhang@ouc.edu.cn

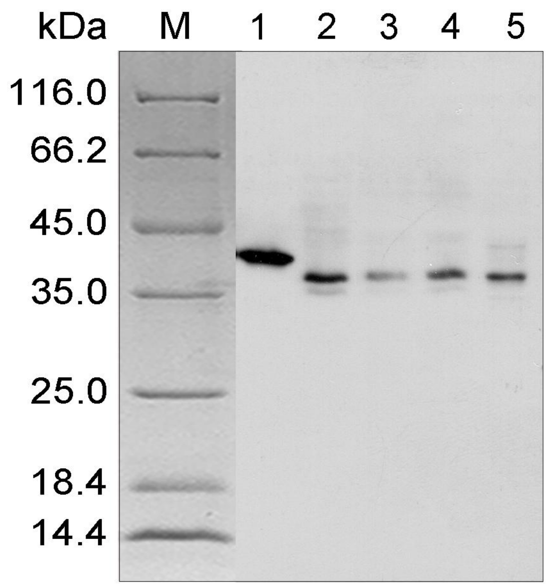

Supplementary Fig. 1 The characterization of the mouse anti-human ELAVL1a antibody reacting with different ELAVL1a. The antibody could not only react with the recombinant zebrafish ELAVL1a protein (lane1), the natural ELAVL1a proteins from zebrafish tissues (skin and muscle, lane2 and 3) and embryos (lane 4), but also react with the natural ELAVL1a proteins from mouse tissue (muscle, lane5). lane M, marker.

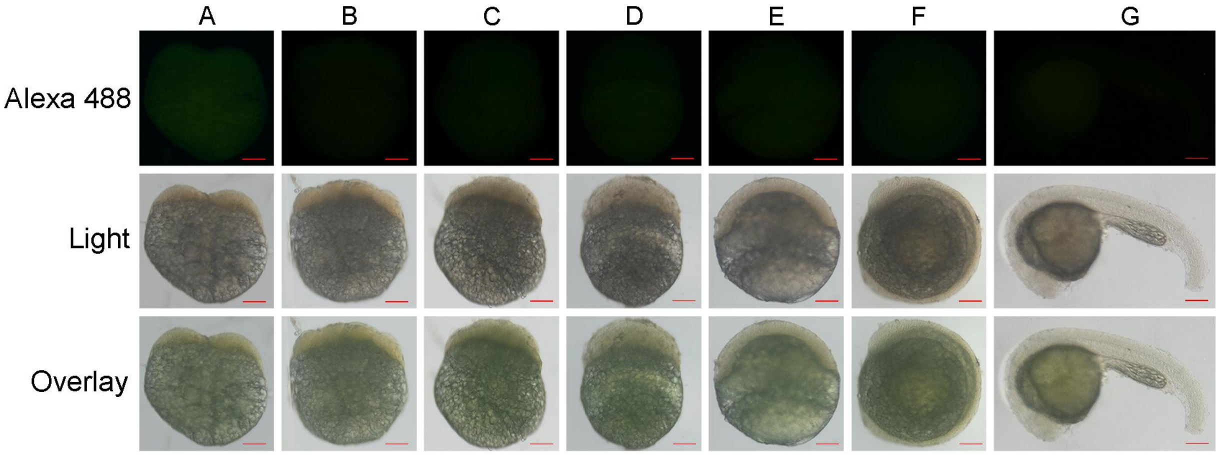

Supplementary Fig. 2 No positive signal in the immunohistochemical assay when using the isotype antibody as control instead of ELAVL1a antibody. (A) 2-cell stage (about 0.75 h); (B) 8-cell stage (~1.25 h); (C) 16-cell stage (~ 1.5 h); (D) 256-cell stage (~2.5 h); (E) 50% epiboly stage (~5.3 h); (F) 10-somite stage (~15 h); (G) 24-h post-fertilization (24-hpf); Scale bars represent 100 μm.

```
GGCACGAGGTTCATTCATCTGCAGATTCGGAGAGACGTTGAAGTCGGAGGTGTACGACATGTCGAACGGT   70
                                                          M  S  N  G
TACGAAGACCACATGGCCGATGAGCTCATCGACTCCAAAACCAACCTTATCGTCAACTACCTGCCACAGA   140
 Y  E  D  H  M  A  D  E  L  I  D  S  K  T  N  L  I  V  N  Y  L  P  Q
ATATGAGCCAGGATGAGCTGCGGAGTCTCTTCAGCAGCATTGGGGAGGTGGAGTCTGCCAAACTTATCCG   210
N  M  S  Q  D  E  L  R  S  L  F  S  S  I  G  E  V  E  S  A  K  L  I  R
AGACAAAGTAGCAGGCCACAGTTTAGGGTACGGATTTGTTAACTATGTTAACCCTAATGATGCAGAAAGA   280
  D  K  V  A  G  H  S  L  G  Y  G  F  V  N  Y  V  N  P  N  D  A  E  R
GCAATCAGTACTCTCAATGGACTGAGACTACAGTCTAAAACTATCAAGGTGTCATATGCCAGGCCAAGCT   350
 A  I  S  T  L  N  G  L  R  L  Q  S  K  T  I  K  V  S  Y  A  R  P  S
CTGACTCCATCAAGGATGCTAATCTTTACATCAGTGGGCTGCCTAAAACAATGACACAAAAGGATGTTGA   420
S  D  S  I  K  D  A  N  L  Y  I  S  G  L  P  K  T  M  T  Q  K  D  V  E
AGAAATGTTCGGACGTTATGGTCGAATAATCAACTCCCGTGTACTTGTTGATCAGGCATCAGGACTCTCT   490
  E  M  F  G  R  Y  G  R  I  I  N  S  R  V  L  V  D  Q  A  S  G  L  S
CGTGGTGTGGCTTTCATTCGATTTGACAAAAGGGCAGAGGCAGAGGACGCAATCAAGGACTTGAATGGAC   560
 R  G  V  A  F  I  R  F  D  K  R  A  E  A  E  D  A  I  K  D  L  N  G
AAAAACCACCAGGCGCTGCTGAGCAGATGACTGTGAAGTTTGCAGCCAGTCCCAACCAAGTGAAAAACAC   630
Q  K  P  P  G  A  A  E  Q  M  T  V  K  F  A  A  S  P  N  Q  V  K  N  T
ACAAGTTATTCCCCAGGTGTATCACCAACAGTCTCGCCGCTTTGGAGGACCCGTCCACCACCAGGCCCAG   700
  Q  V  I  P  Q  V  Y  H  Q  Q  S  R  R  F  G  G  P  V  H  H  Q  A  Q
AGATTCAGGTTTTCTCCGATGAGCGTGGACCACATGAGCGGCATGTCTGGTGTAAACGTGCCTGGAAACT   770
 R  F  R  F  S  P  M  S  V  D  H  M  S  G  M  S  G  V  N  V  P  G  N
CTTCATCAGGCTGGTGCATCTTCATCTACAACTTGGGCCAGGACGCAGATGAGGGCATTCTGTGGCAGAT   840
S  S  S  G  W  C  I  F  I  Y  N  L  G  Q  D  A  D  E  G  I  L  W  Q  M
GTTCGGGCCCTTCGGCGCAGTCACAAACGTCAAAGTTATCCGTGACTTCAACACCAACAAGTGCAAAGGA   910
  F  G  P  F  G  A  V  T  N  V  K  V  I  R  D  F  N  T  N  K  C  K  G
TTTGGGTTTGTTACCATGACACACTACGAAGAGGCCGCCATGGCTATCGCCAGTCTCAACGGCTACCGTC   980
 F  G  F  V  T  M  T  H  Y  E  E  A  A  M  A  I  A  S  L  N  G  Y  R
TCGGGGACAAGATTTTACAAGTGTCATTCAAAACAAGCAAGTCGCACAAGTAGAAAGCAGGGTTTGCCTG   1050
L  G  D  K  I  L  Q  V  S  F  K  T  S  K  S  H  K  *
CAAAATGAAGGAGAGAGGGGGGGAGGAAGGGAGGGTTTTTCTGCGATGCCAGAGAAATTGGCAGTTTTG   1120
AAAAACTATTATTTAGTGTATAATCAAGTCTTTGCAAATGTTCACGTTTTCTCAGTCTCGTTTAGACAT   1190
CTGTTTGACAGGTTATTTTTGTTTCTTTTAAGATTTAAGCTGTTTGATGCTTCAAATTCAAGTGTTTTAG   1260
GTGTTCTGGTTAATCCCAATATTTGACCAATTTCGCATTTGATAAAAATTTATCTCCGATGTTAGCCTGA   1330
TATTGTTGCTACGTGCCATATGTGGTCGGACGCCTCATTCGCCAGATGCTGGATTTAAAACTTGAGATTT   1400
TAAGTTTTTTAATCTTTCGCCAGTATGTACATCCTACATTGTTCTTCAGTATCTACAATGCCTTAATTTG   1470
ATGACGGTTGTCCTCTTTGCTCTCAATAATGTAGCACTTTTTGGTTTGGCAGGAAGCTCCTTGCTCATCT   1540
CACAACAGATCATTCGTTCATTAAAATCCGAGTTTTAAATCCAGCATGCTGAGTGTTTCACAGAAAAGAA   1610
GAGAGGCAGTGGTAAAGGAACCCGAGTGTCTATGAAATGCCTGTTGTCAGTGAGGGGAAAGTCTTTAACT   1680
AACGTGTTACTTGTTTGTAAAGGGACCCTCTGAAAAGACTGATATCTTAGAAGGACCAAAGACATGAATG   1750
GAAAGTTTAGTTTCTCCCCGTCAGAGGTGCAGCTTGCACATCTCTGAGCATATTCCAAAAATATATTTGG   1820
ACATCTAAATACTAGGTACTGAGTATTTATGTAAAAATGTTATTACATTTGAGCATCAGCATCTTCTGTT   1890
TATGGTTTGCATTTTTTTGTTTTGTTTTTCTCTAGAGTAAAACATACTTTGAGTTTAGTTTGGTTCTTTG   1960
AAAATAGATATACAGTACTAATGTGTATGCTTTTTAAGACTGCCTTGTGATTTAATTTGTGTTGTTCCA   2030
TTTAGTTTTTTTTCTTTTTGTTAATTTGCTTTTGTCTTTCCTTTTGAAACCTTTCTTTTTGTTTCACAT   2100
GGCATTTACAATCATTAAATTTTATTTCGTTTTGCTCAAAAAAAAAAAAAAAAAAAAAA   2160
```

Supplementary Fig. 3 The full-length cDNA sequence and deduced amino acid sequence of zebrafish *elavl1a*. Nucleotides are numbered in the 5'-3' direction. The 5'- UTR is red and the 3'-UTR is green. The derived amino acid sequence is shown underneath the nucleotide sequence using single-letter codes. The stop codon is indicated by an asterisk.

Percent identity (upper triangle); **Divergence** (lower triangle)

	1	2	3	4	5	6	7	8	9	10	11	12	13	14	15	16	17	18	19	20	21	22		
**1**		100.0	100.0	98.8	98.5	98.5	99.4	99.4	98.5	98.8	97.9	92.9	92.3	92.3	85.5	86.2	83.4	84.9	83.5	67.6	38.6	86.4	**1**	*Homo sapiens* ELAVL1
**2**	0.0		100.0	98.8	98.5	98.5	99.4	99.4	98.5	98.8	97.9	92.9	92.3	92.3	85.5	86.2	83.4	84.9	83.5	67.6	38.6	86.4	**2**	*Pongo abelii* ELAVL1
**3**	0.0	0.0		98.7	98.7	98.7	99.3	99.3	98.7	99.0	97.7	93.1	93.1	92.5	86.8	87.5	84.5	86.2	84.4	69.0	39.7	88.8	**3**	*Pan troglodytes* ELAVL1
**4**	1.2	1.2	1.3		98.5	98.5	98.8	98.8	97.9	98.2	97.2	92.3	91.7	91.7	84.6	85.2	82.5	84.0	83.2	67.6	38.3	85.5	**4**	*Oryctolagus cuniculus* ELAVL1
**5**	1.6	1.6	1.3	1.6		100.0	98.5	98.5	97.5	97.9	96.9	93.3	92.6	92.0	85.5	86.2	83.4	84.9	83.5	67.0	38.6	86.4	**5**	*Mus musculus* ELAVL1
**6**	1.6	1.6	1.3	1.6	0.0		98.5	98.5	97.5	97.9	96.9	93.3	92.6	92.0	85.5	86.2	83.4	84.9	83.5	67.0	38.6	86.4	**6**	*Rattus norvegicus* ELAVL1
**7**	0.6	0.6	0.7	1.2	1.6	1.6		100.0	98.8	99.1	97.9	92.9	92.3	92.3	85.5	86.2	83.4	84.9	83.2	67.3	38.6	86.1	**7**	*Bos Taurus* ELAVL1
**8**	0.6	0.6	0.7	1.2	1.6	1.6	0.0		98.8	99.1	97.9	92.9	92.3	92.3	85.5	86.2	83.4	84.9	83.2	67.3	38.6	86.1	**8**	*Ovis aries* ELAVL1
**9**	1.6	1.6	1.3	2.2	2.5	2.5	1.2	1.2		99.7	97.5	92.6	92.0	92.0	85.5	86.2	83.4	84.0	82.6	66.7	38.6	85.5	**9**	*Gallus gallus* ELAVL1
**10**	1.2	1.2	1.0	1.9	2.2	2.2	0.9	0.9	0.3		97.9	92.9	92.3	92.3	85.8	86.5	83.7	84.3	82.9	67.0	38.6	85.8	**10**	*Zonotrichia albicollis* ELAVL1
**11**	2.2	2.2	2.3	2.8	3.1	3.1	2.2	2.2	2.5	2.2		91.7	91.1	91.1	85.8	86.5	83.7	84.9	83.5	67.0	38.6	86.1	**11**	*Pogona vitticeps* ELAVL1
**12**	7.4	7.4	7.2	8.1	7.1	7.1	7.4	7.4	7.8	7.4	8.8		97.5	97.2	82.8	83.4	81.5	83.1	83.5	65.1	38.3	83.6	**12**	*Xenopus tropicalis* ELAVL1
**13**	8.1	8.1	7.2	8.8	7.8	7.8	8.1	8.1	8.4	8.1	9.5	2.5		98.5	83.4	83.4	81.8	83.7	84.2	64.8	38.3	83.3	**13**	*Xenopus laevis* ELAVL1-B
**14**	8.1	8.1	8.0	8.8	8.4	8.4	8.1	8.1	8.4	8.1	9.5	2.8	1.6		83.1	83.1	80.9	82.8	83.9	65.4	38.6	83.0	**14**	*Xenopus laevis* ELAVL1-A
**15**	16.1	16.1	14.5	17.3	16.1	16.1	16.1	16.1	16.1	15.7	15.7	19.6	18.8	19.2		98.0	91.9	91.7	82.7	66.2	38.1	87.0	**15**	*Mastacembelus armatus* ELAVL1
**16**	15.3	15.3	13.7	16.5	15.3	15.3	15.3	15.3	15.3	15.0	15.0	18.8	18.8	19.2	2.0		93.3	93.1	82.9	66.2	38.1	88.0	**16**	*Seriola lalandi dorsalis* ELAVL1
**17**	18.8	18.8	17.4	20.0	18.8	18.8	18.8	18.8	18.8	18.4	18.4	21.2	20.8	22.1	8.6	7.0		93.3	82.0	64.6	37.8	86.1	**17**	*Nothobranchius furzeri* ELAVL1
**18**	16.9	16.9	15.3	18.0	16.9	16.9	16.9	16.9	18.0	17.7	16.9	19.2	18.4	19.6	8.9	7.2	7.0		83.0	64.6	38.1	88.6	**18**	*Xipophorus maculatus* ELAVL1
**19**	18.6	18.6	17.6	19.0	18.6	18.6	19.0	19.0	19.8	19.4	18.6	18.6	17.8	18.2	19.7	19.5	20.7	19.3		63.8	38.1	85.7	**19**	*Danio rerio* ELAVL1b
**20**	42.3	42.3	39.9	42.3	43.4	43.4	42.8	42.8	43.9	43.4	43.4	46.7	47.3	46.2	44.8	44.8	47.6	47.6	49.2		38.8	65.3	**20**	*Branchiostoma belcheri* ELAVL1
**21**	116.9	116.9	112.8	118.2	116.9	116.9	116.9	116.9	116.9	116.9	116.9	118.2	118.2	116.9	118.8	118.8	120.1	118.8	119.2	116.1		38.6	**21**	*Stylophora pistillata* ELAVL1
**22**	15.0	15.0	12.2	16.2	15.0	15.0	15.4	15.4	16.2	15.8	15.4	18.5	18.9	19.3	14.3	13.2	15.4	12.4	15.9	46.4	116.9		**22**	*Danio rerio* ELAVL1a
	**1**	**2**	**3**	**4**	**5**	**6**	**7**	**8**	**9**	**10**	**11**	**12**	**13**	**14**	**15**	**16**	**17**	**18**	**19**	**20**	**21**	**22**		

Supplementary Fig. 4 Amino acid sequence identity of zebrafish ELAVL1a with other species ELAVL1 proteins. The amino acid identity was calculated using the Clustal W program within the MegAlign of the DNASTAR software package (version 5.0). The sequence comparison showed that zebrafish ELAVL1a shares 85.5% ~ 88.8%, 85.5% ~ 85.8%, 86.1%, 83.0% ~ 83.6%, 85.7% ~ 88.0%, 65.3% and 38.6% identical to mammalian, avian, reptile, amphibian, fish, amphioxus and coral counterparts. The accession numbers of ELAVL1 proteins used are: *Homo sapiens* ELAVL1 (NP_001410.2), *Pan troglodytes* ELAVL1 (NP_001267123.1) , *Pongo abelii* ELAVL1 (XP_024092738.1), *Oryctolagus cuniculus* ELAVL1 (NP_001172077.1), *Mus musculus* ELAVL1 (NP_034615.2), *Rattus norvegicus* ELAVL1 (NP_001102318.1), *Bos taurus* ELAVL1 (NP_001069922.1), *Ovis aries* ELAVL1 (NP_001159666.1), *Gallus gallus* ELAVL1 (NP_990164.1), *Zonotrichia albicollis* ELAVL1 (XP_014127634.1), *Pogona vitticeps* ELAVL1 (XP_020636529.1), *Xenopus tropicalis* ELAVL1 (NP_001005461.1), *Xenopus laevis* ELAVL1 (NP_001084078.1), *Xenopus laevis* ELAVL1 (NP_001088628.1), *Mastacembelus*

armatus ELAVL1 (XP_026175681.1), *Seriola lalandi dorsalis* ELAVL1 (XP_023286570.1), *Nothobranchius furzeri* ELAVL1 (SBP43464.1), *Xiphophorus maculatus* ELAVL1 (XP_005809401.1), *Danio rerio* ELAVL1a (NP_571527.1), *Danio rerio* ELAVL1b (NP_001295543.1), *Branchiostoma belcheri* ELAVL1 (BAB62225.1), *Stylophora pistillata* ELAVL1 (PFX27751.1).

Percent identity

Divergence	1	2	3	4	5		
1		81.4	81.7	65.6	69.8	1	*Dnio rerio* ELAVL2
2	21.4		86.0	67.4	69.8	2	*Dnio rerio* ELAVL3
3	21.0	15.5		65.6	70.4	3	*Dnio rerio* ELAVL4
4	45.8	42.7	45.8		85.7	4	*Dnio rerio* ELAVL1b
5	38.7	38.7	37.7	15.9		5	*Dnio rerio* ELAVL1a
	1	2	3	4	5		

Supplementary Fig. 5 Amino acid sequence identity of zebrafish ELAVL1a, ELAVL1b, ELAVL2, ELAVL3 and ELAVL4. The amino acid identity was calculated using the Clustal W program within the MegAlign of the DNASTAR software package (version 5.0). The sequence comparison showed that the ELAVL1a was 85.7%, 69.8%, 69.8% and 70.4% identical to ELAVL1b, ELAVL2, ELAVL3 and ELAVL4The accession numbers of proteins used are: *Danio rerio* ELAVL2 (NP_001002172.2); *Danio rerio* ELAVL3 (NP_571524.1); *Danio rerio* ELAVL4 (NP_001231529.1); *Danio rerio* ELAVL1b (NP_001295543.1); *Danio rerio* ELAVL1a (NP_571527.1).

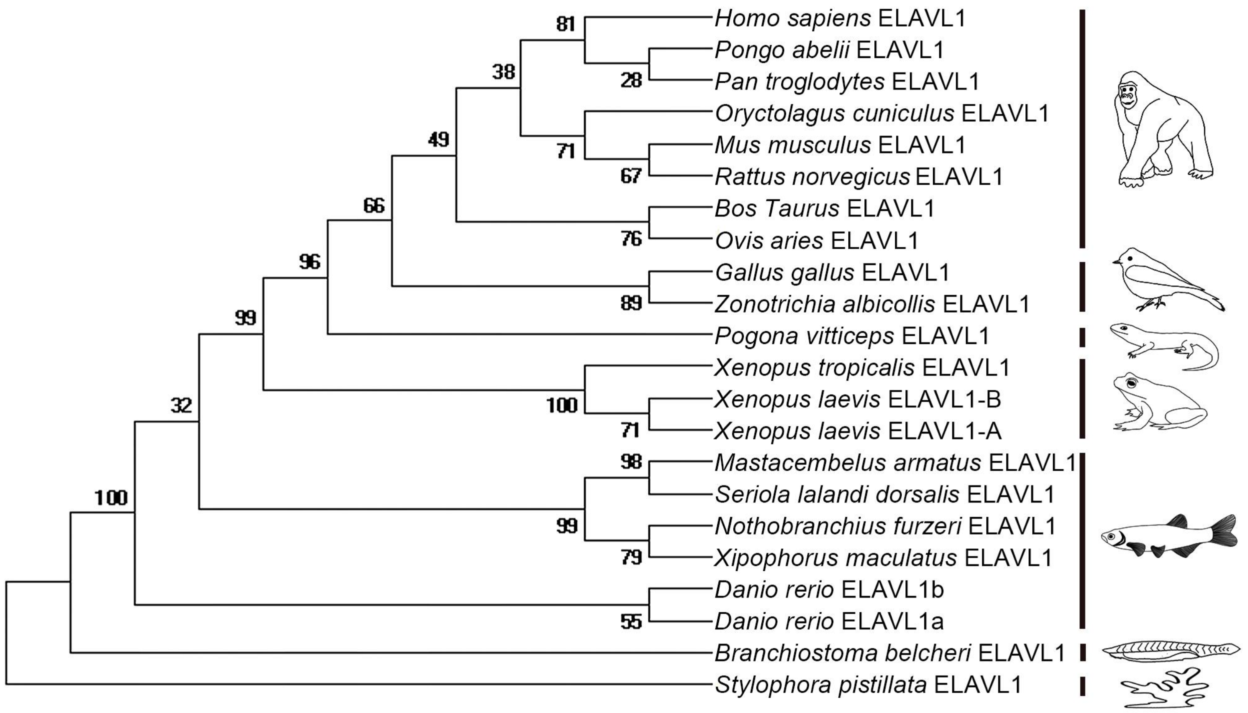

Supplementary Fig.6 Phylogenetics of zebrafish ELAVL1a. Phylogenetic tree constructed by MEGA, version 7.0, using the neighbor-joining method. The accession numbers of proteins used are listed in Supplementary Fig. 4 legend. All stick figures of animal were drawn by Shousheng Ni (first author) using the Procreate software (version 5.1.5).

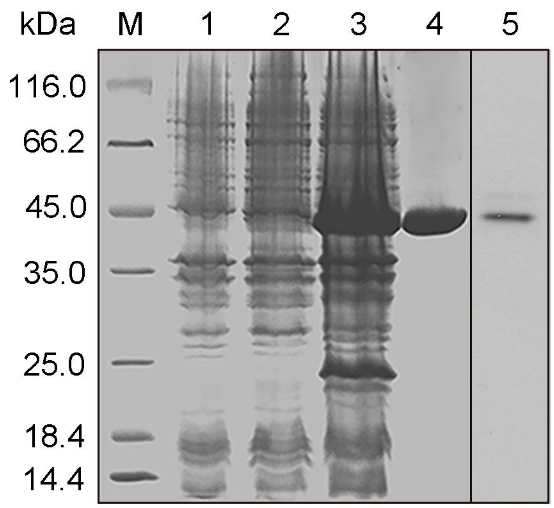

Supplementary Fig. 7. SDS-PAGE of recombinant protein rELAVL1a. Lane M, marker; lane 1, negative control (empty vector *pET28a*); lane 2 and 3, total cellular extracts from *E. coli* BL21 containing expression vector *pET28a/elavl1a* before induction (2) or induced with IPTG (3); lane 4, purified rELAVL1a; lane 5, Western blotting of rELAVL1a.

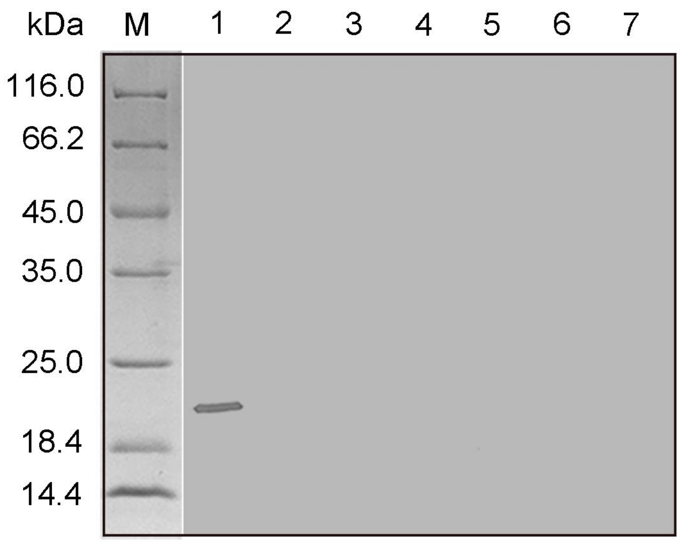

Supplementary Fig. 8. No affinity of TRX-His-tag peptide with the Gram-positive and Gram-negative bacteria. Lane M, marker; lane 1, purified TRX-His-tag; lane 2, 3, 4, 5, 6 and 7, *M. luteus*, *B. subtilis*, *S. aureus*, *E. coli*, *V. anguillarum* and *A. hydrophila* incubated in the presence of TRX-His-tag peptide.

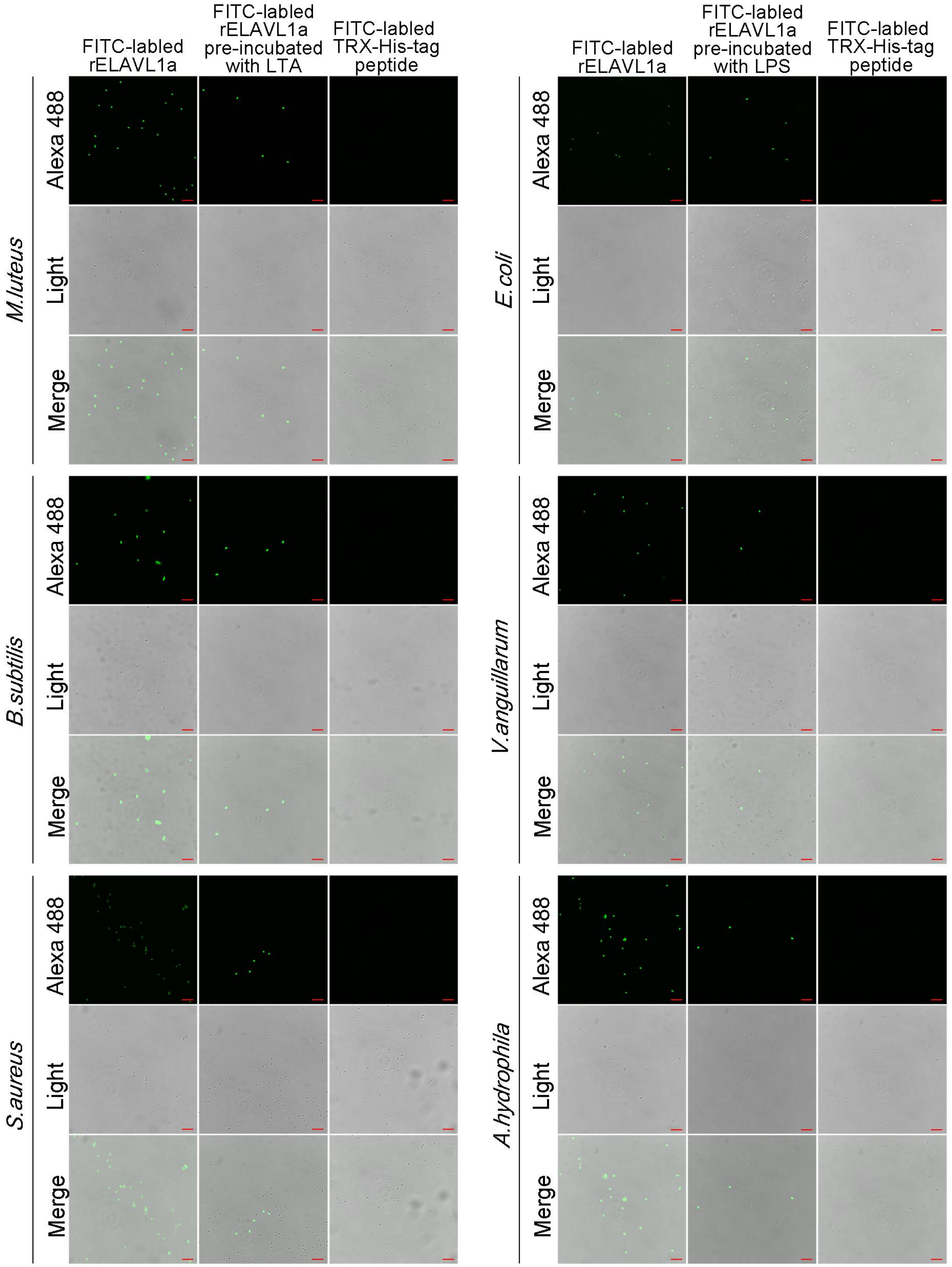

Supplementary Fig. 9 Binding of FITC-labeled rELAVL1a to Gram-positive and -negative bacteria. Scale bars represent 10 μm.

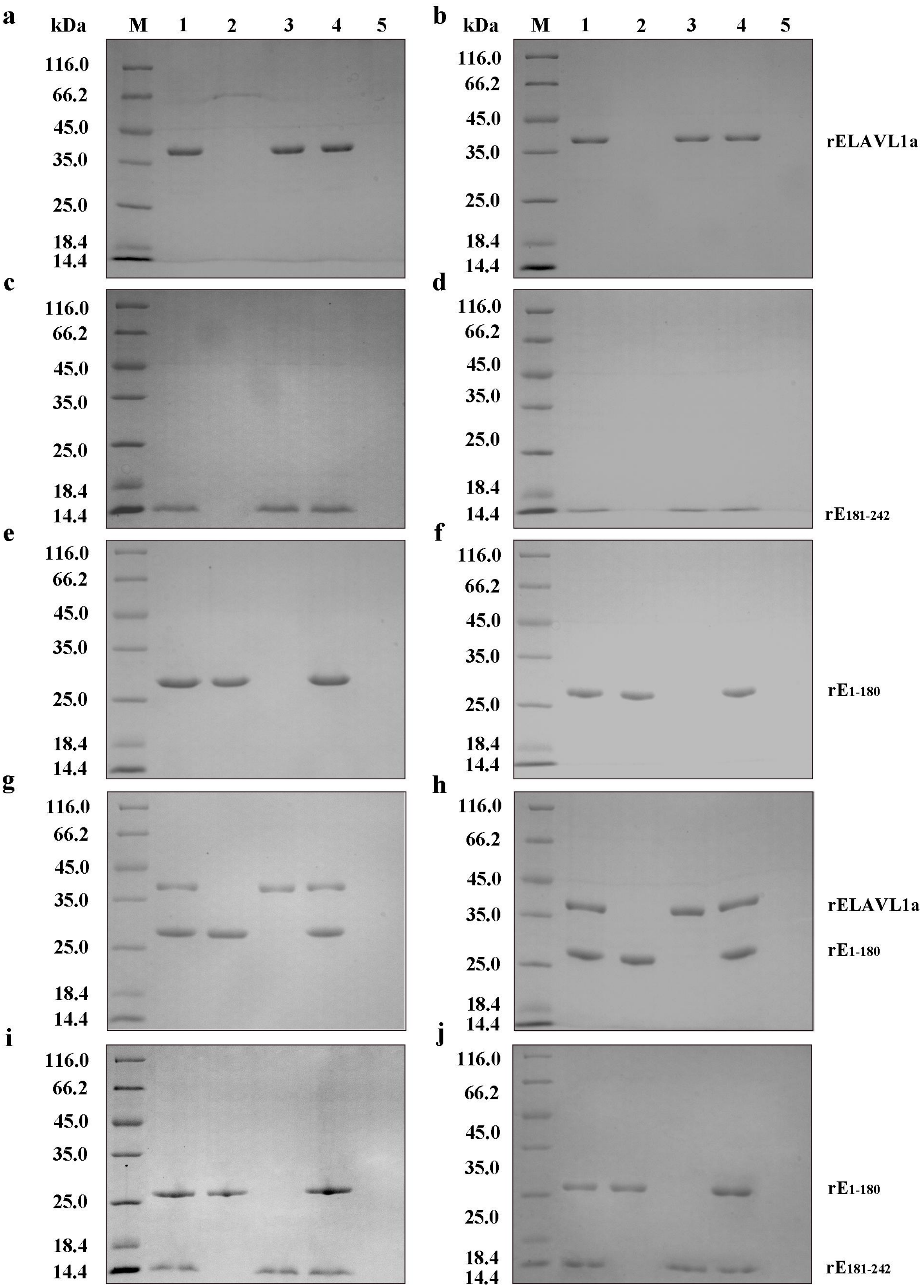

Supplementary Fig. 10. Pull-down between the ELAVL1a, $E_{181-242}$ and E_{1-180} proteins and

LTA or LPS. a-f Pull-down experiment between the ELAVL1a, $E_{181-242}$ and E_{1-180} proteins and LTA (a, c and e) or LPS (b, d and f); **g-j** Competitive binding assays by introducing the E_{1-180} protein in the experiment between the rELAVL1a protein and LTA (**g**) or LPS (**h**), $E_{181-242}$ and LTA (**i**) or LPS (**j**). lane M, Marker; lane 1, the samples before loaded onto resin column; lane 2, effluent fractions after initial buffer (10 mM Tris-HCl with 150mM NaCl, pH7.4) washing; lane 3, effluent fractions after elution buffer (4 M Urea in 10 mM Tris-HCl, pH7.4) washing; lane 4, effluent fractions after initial buffer washing from affinity resin without LTA or LPS conjugated; lane 5, effluent fractions after elution buffer washing from LTA or LPS-conjugated affinity resin loaded with recombinant TRX-His tag.

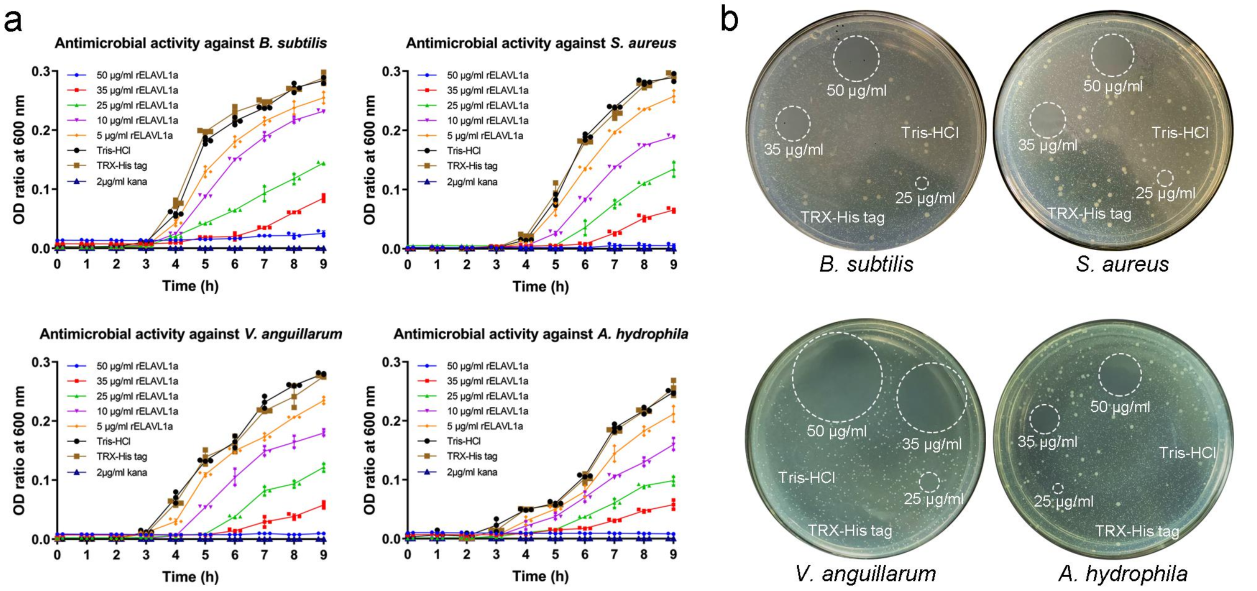

Supplementary Fig. 11. Antibacterial activity of rELAVL1a. a Antibacterial activities of rELAVL1a against Gram-positive bacteria *B. subtilis* and *S. aureus* and Gram-negative bacteria *V. anguillarum* and *A. hydrophila*. Each point in the graph represents the mean ± S.D. (n = 3). The data are from three independent experiments performed in triplicate. The bars represent the mean ± S.D. **b** Antibacterial activities of rELAVL1a against Gram-positive bacterial *B. subtilis* and *S. aureus* and Gram-negative bacteria *V. anguillarum* and *A. hydrophila* by the agar diffusion test method. The halo without the cup (bottom) are shown.

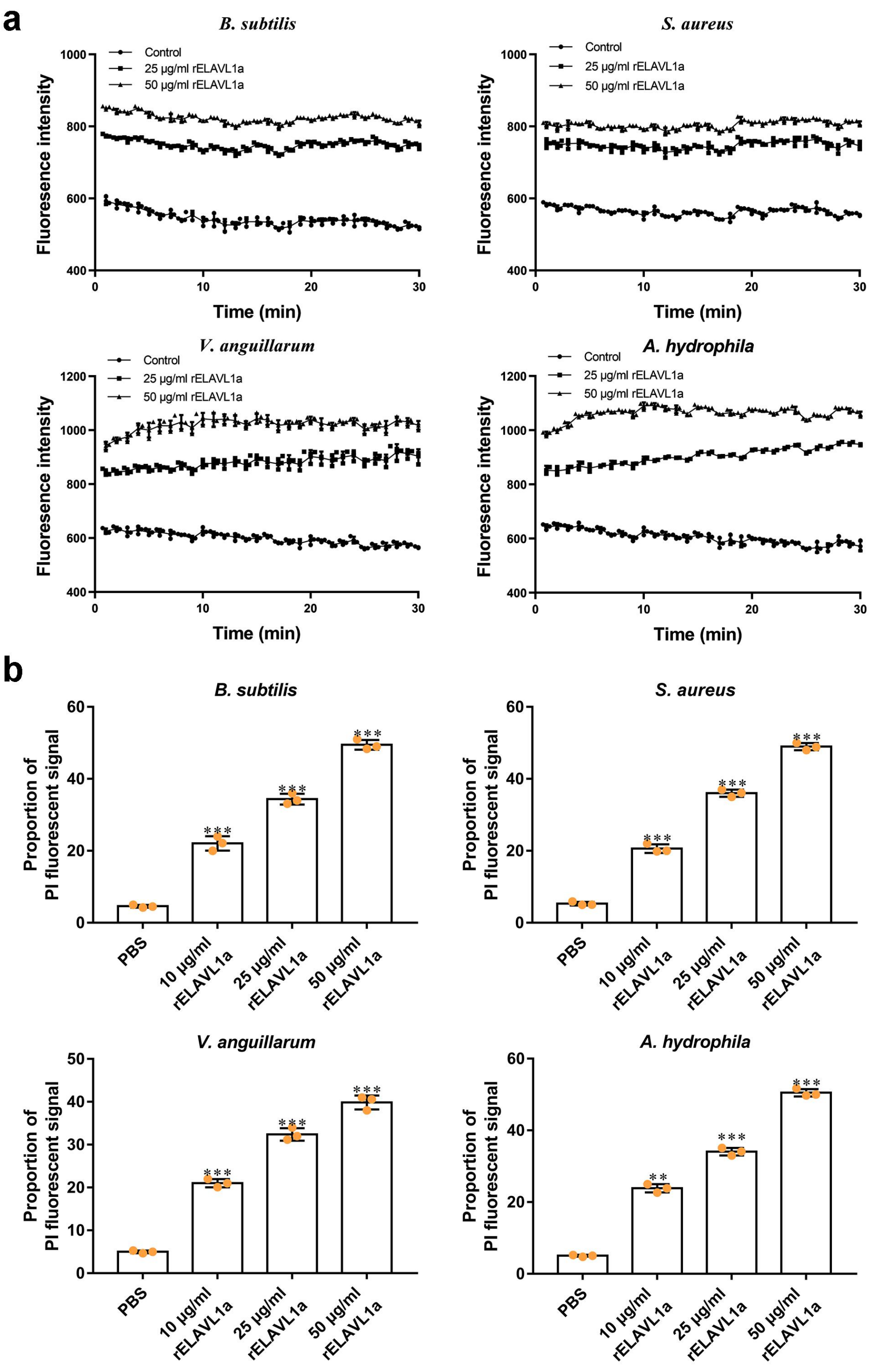

Supplementary Fig. 12. **rELAVL1a was able to disrupt the bacterial membranes by a membrane lytic mechanism including a combined action of membrane depolarization and membrane permeabilization. a** The rELAVL1a caused depolarization of the bacterial plasma

membrane. The changes in fluorescence intensity were recorded with a Tecan GENios plus spectrofluorometer at an excitation wavelength of 622 nm and an emission wavelength of 670 nm. **b** The effects of rELAVL1a on the membrane integrity of *B. subtilis*, *S. aureus*, *V. anguillarum* and *A. hydrophila,* cells analyzed by flow cytometry. All data were expressed as mean values ± S.D. (n = 3). The data are from three independent experiments performed in triplicate. The bars represent the mean ± S.D. The significance of difference was determined by one-way ANOVA. $^{**}p < 0.01$, $^{***}p < 0.001$.

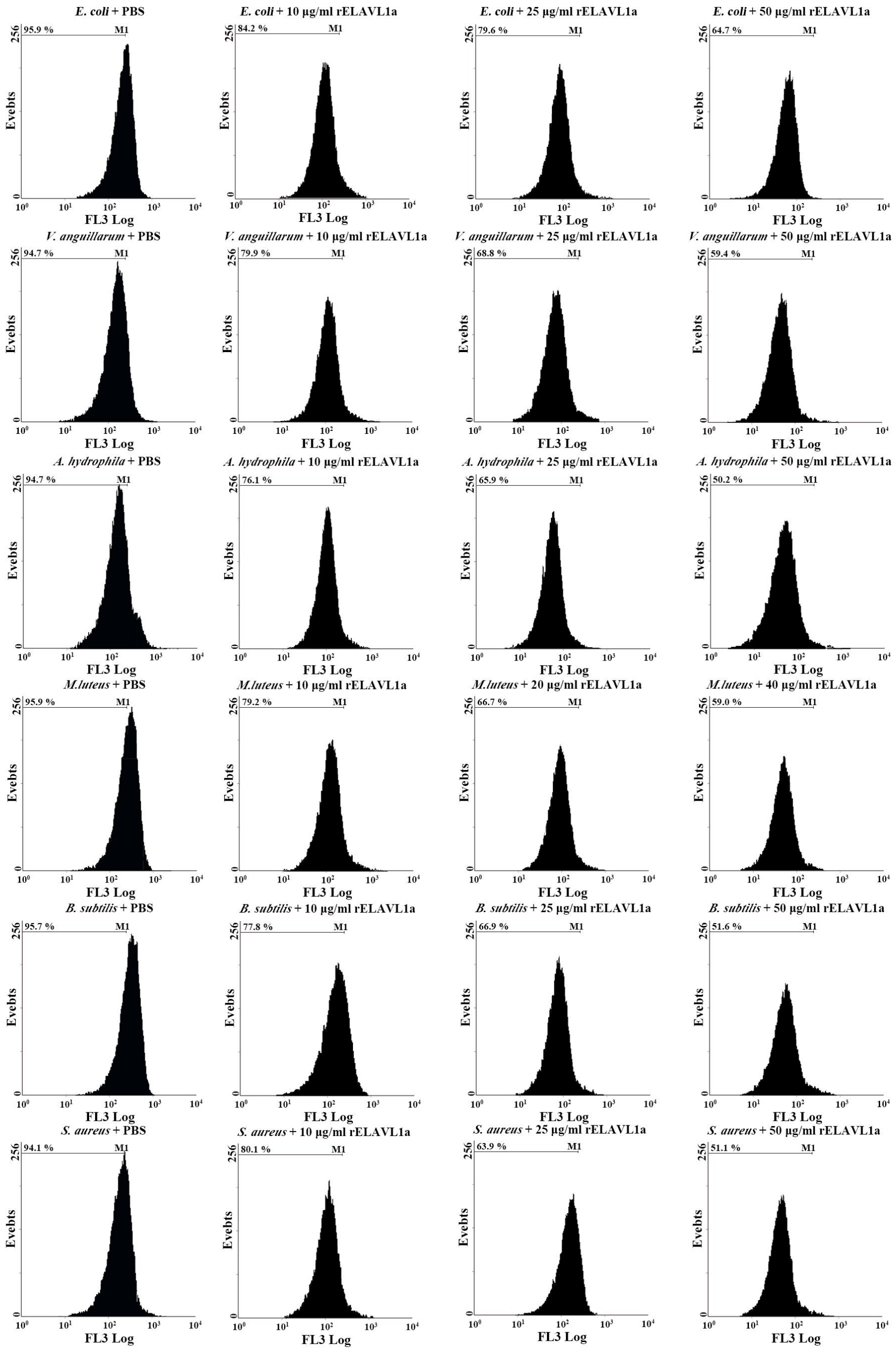

Supplementary Fig. 13 The effects of rELAVL1a on the membrane integrity of *E. coli, V. anguillarum, A. hydrophila, S. aureus*, *B. subtilis* and *M. luteu* cells analyzed by flow cytometry. Data were analyzed using WinMDI, version 2.9, software. The proportion of integrated cells is shown near the marker (M1).

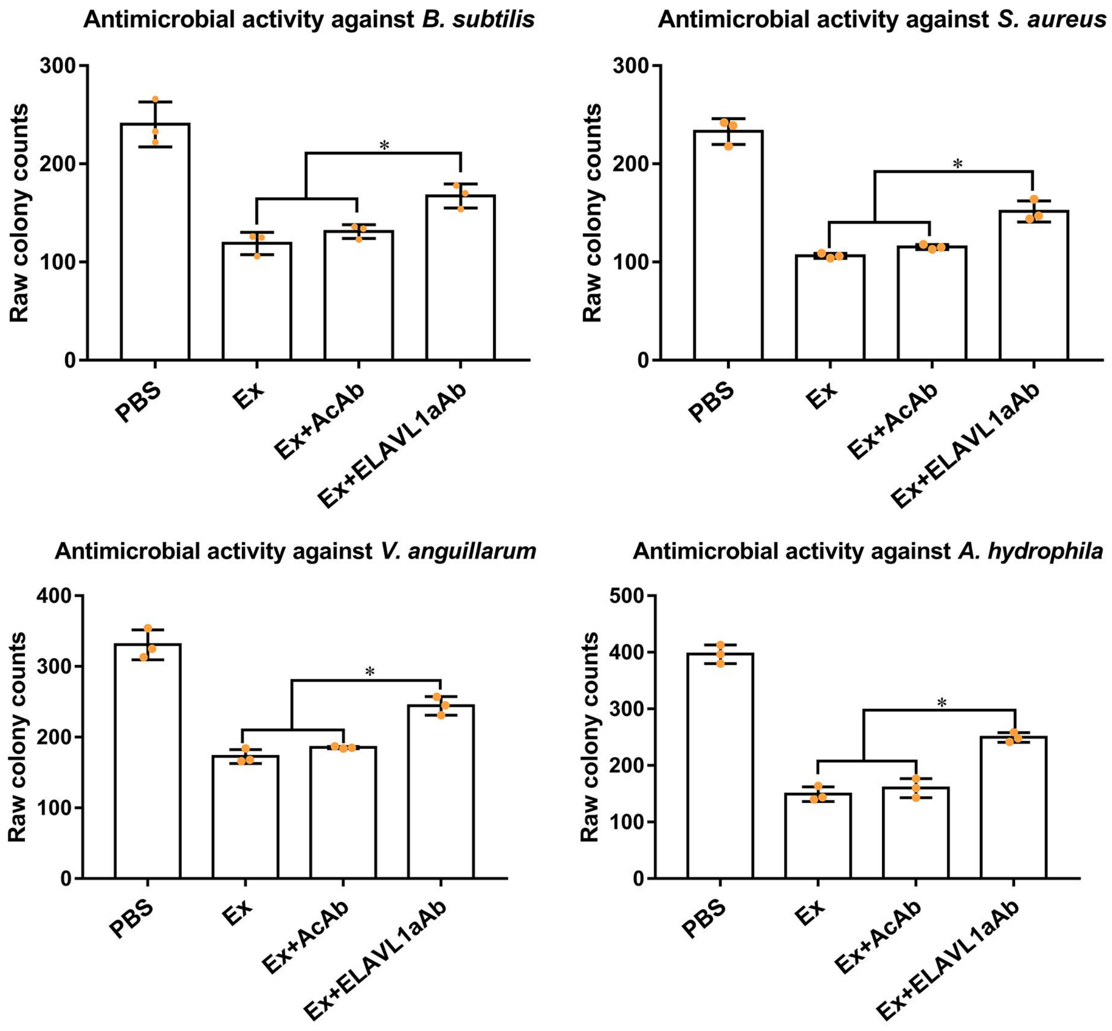

Supplementary Fig. 14 Antimicrobial activities of the embryo extract against *B. subtilis*, *S. aureus*, *V. anguillarum* and *A. hydrophila.* All data were expressed as mean values ± S.D. (n = 3). The data are from three independent experiments performed in triplicate. The bars represent the mean ± S.D. The significance of difference was determined by one-way ANOVA. *, $p < 0.05$. Ex, embryo extract; AcAb, anti-β-actin antibody; ELAVL1aAb, mouse anti-ELAVL1a antibody.

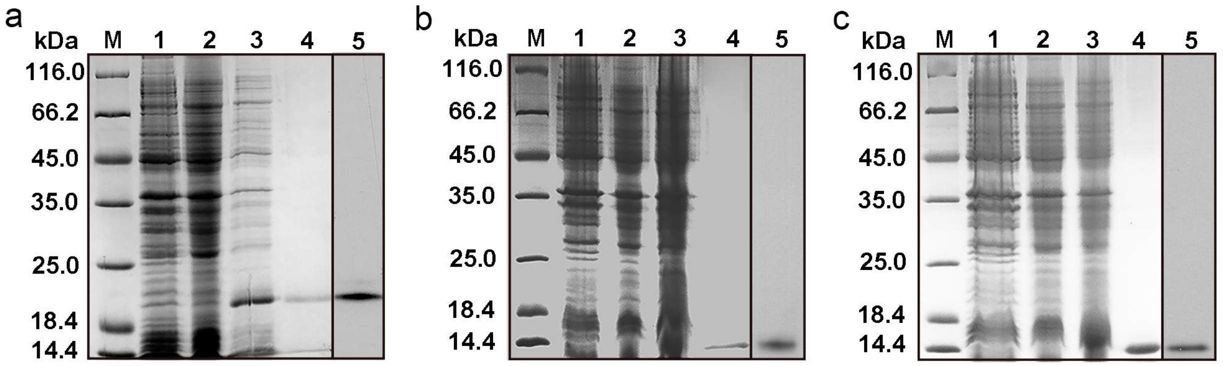

Supplementary Fig. 15 SDS-PAGE and Western blotting of rE$_{1\text{-}180}$ (a), rE$_{181\text{-}242}$ (b) and rE$_{243\text{-}324}$ (c). lane M, marker; lane 1, total cellular extracts from *E. coli* BL21 containing expression vector *pET28a/e1-180* (**a**), *pET28a/e181-242* (**b**) and *pET28a/e243-324* (**c**) before induction; lane 2, total cellular extracts from IPTG-induced *E. coli* BL21 containing expression vector *pET28a/e1-180* (**a**), *pET28a/e181-242* (**b**) and *pET28a/e243-324* (**c**); lane 3, purified rE$_{1\text{-}180}$ (**a**), rE$_{181\text{-}242}$ (**b**) and rE$_{243\text{-}324}$ (**c**); lane 4, Western blotting of rE$_{1\text{-}180}$ (**a**), rE$_{181\text{-}242}$ (**b**) and rE$_{243\text{-}324}$ (**c**).

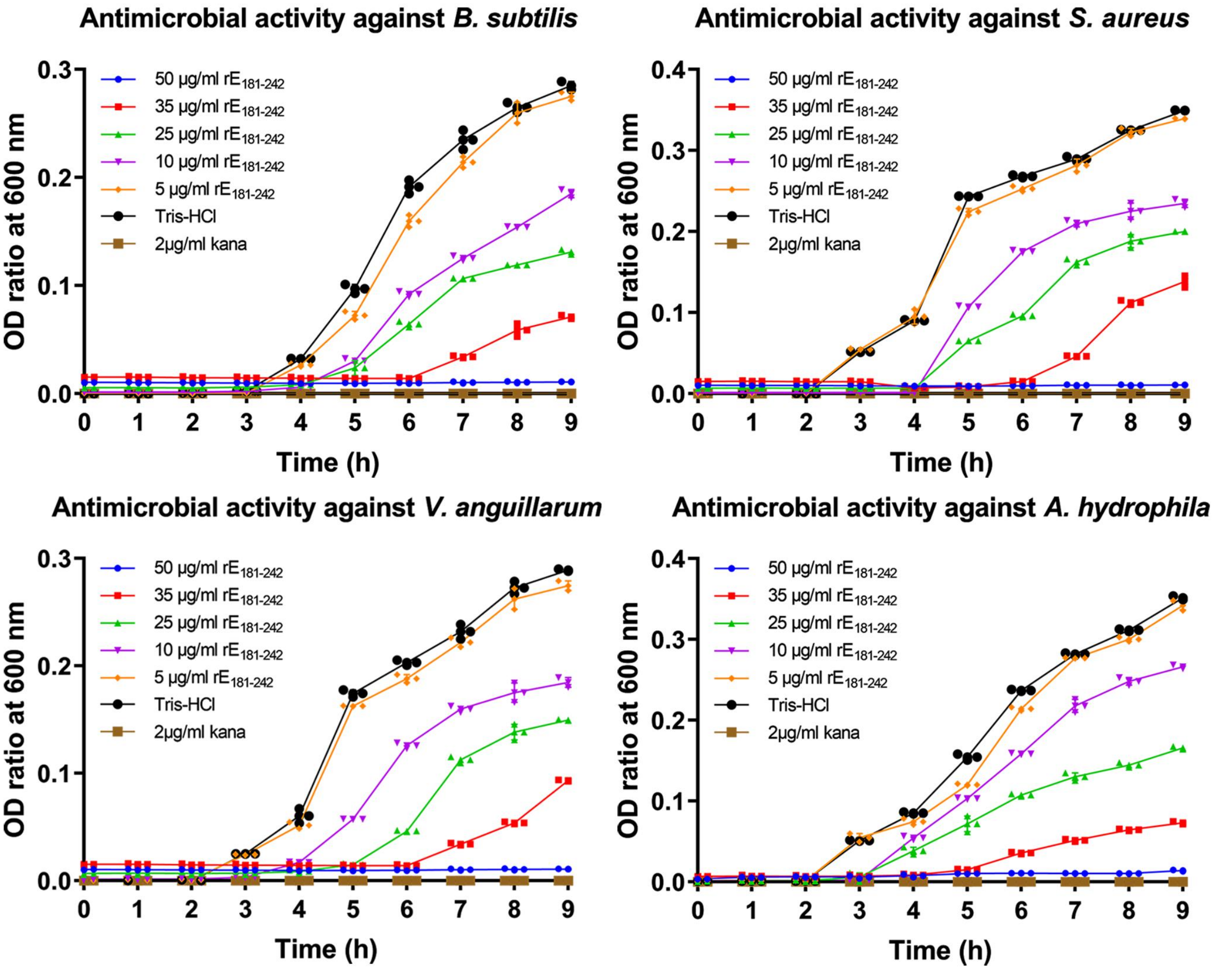

Supplementary Fig. 16 Antibacterial activity of rE$_{181\text{-}242}$ against *B. subtilis*, *S. aureus*, *V. anguillarum*, and *A. hydrophila*. Each point in the graph represents the mean ± S.D. (n = 3). The data are from three independent experiments performed in triplicate. The bars represent the mean ± S.D.

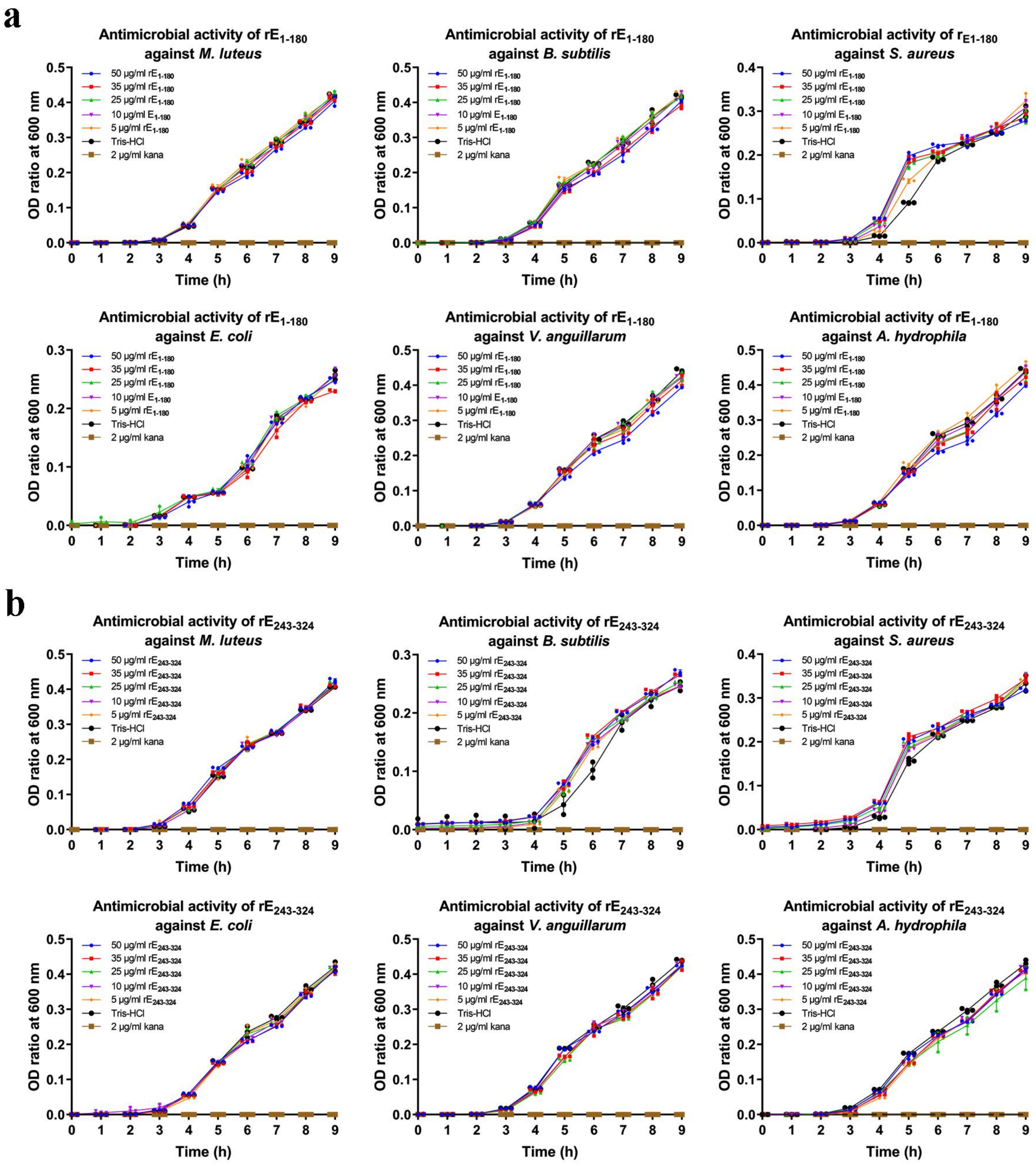

Supplementary Fig. 17 Antibacterial activity of rE$_{1-180}$ and rE$_{243-324}$ against *M. luteus*, *B. subtilis*, *S. aureus*, *E. coli*, *V. anguillarum* and *A. hydrophila*. a Little antibacterial activity of rE$_{1-180}$ against against *M. luteus*, *B. subtilis*, *S. aureus*, *E. coli*, *V. anguillarum* and *A. hydrophila*. **b** Little antibacterial activity of rE$_{243-324}$ against *M. luteus*, *B. subtilis*, *S. aureus*, *E. coli*, *V.*

anguillarum and *A. hydrophila*. Each point in the graph represents the mean ± S.D. (n = 3). The data are from three independent experiments performed in triplicate. The bars represent the mean ± S.D.

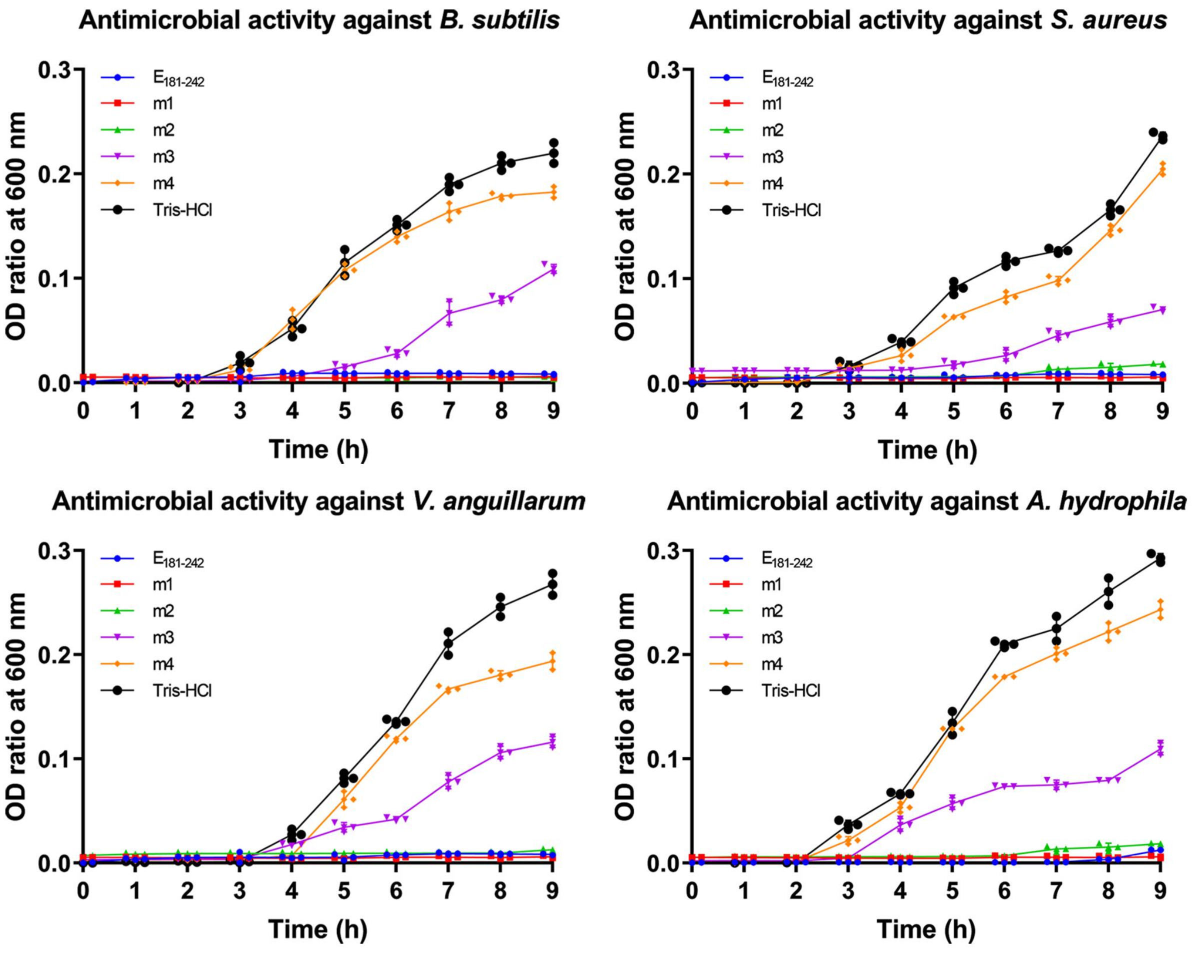

Supplementary Fig. 18 Antibacterial activity of rE$_{181-242}$ mutans against *M. luteus* and *E. coli*. rE$_{181-242}$ was used as positive control and Tris-HCl buffer was used as negative control. Each point in the graph represents the mean ± S.D. (n = 3). The data are from three independent experiments performed in triplicate. The bars represent the mean ± S.D.

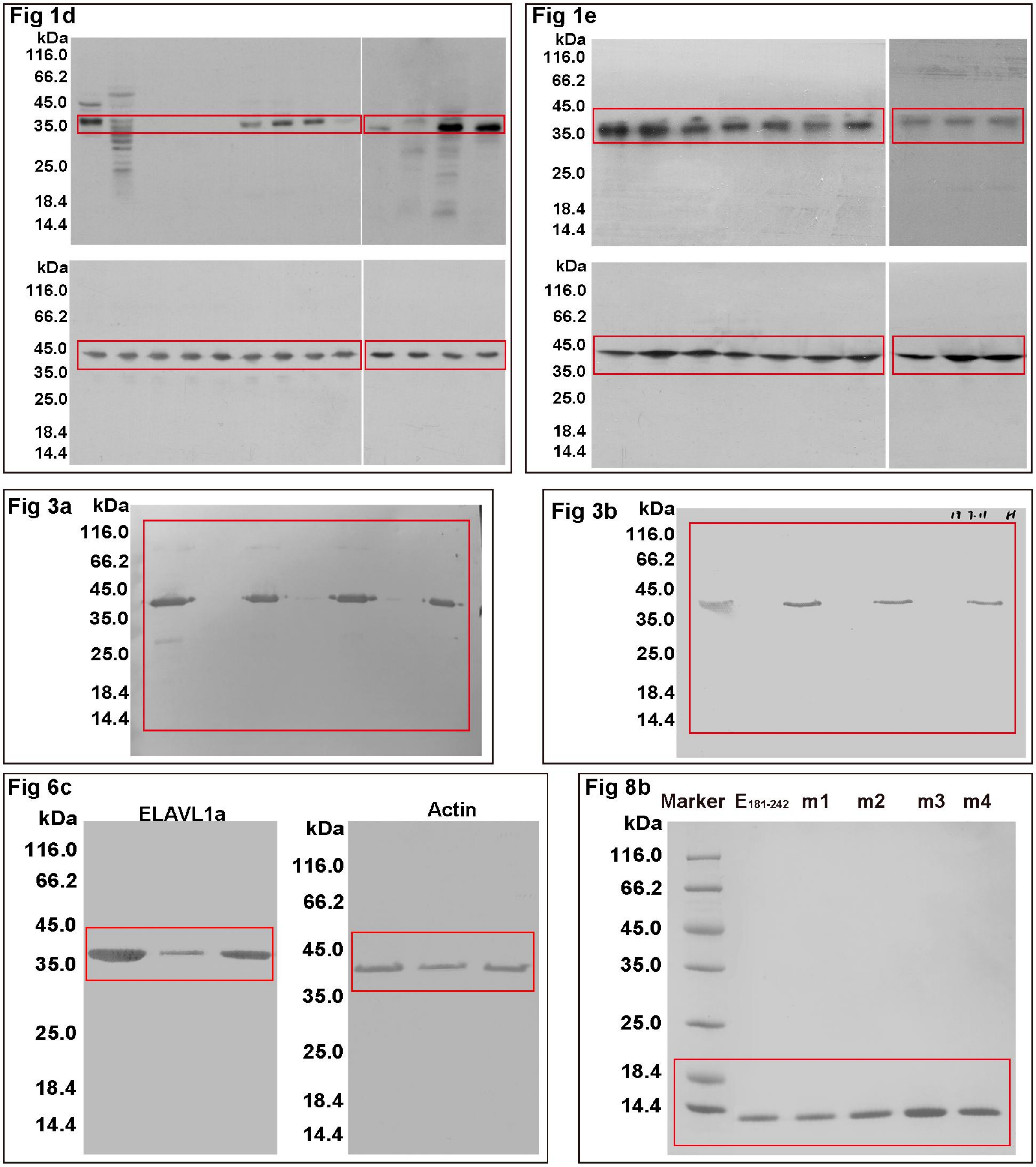

Supplementary Fig. 19 Unprocessed original blot images represented in Fig.1, Fig. 3, Fig. 6 and Fig. 8.

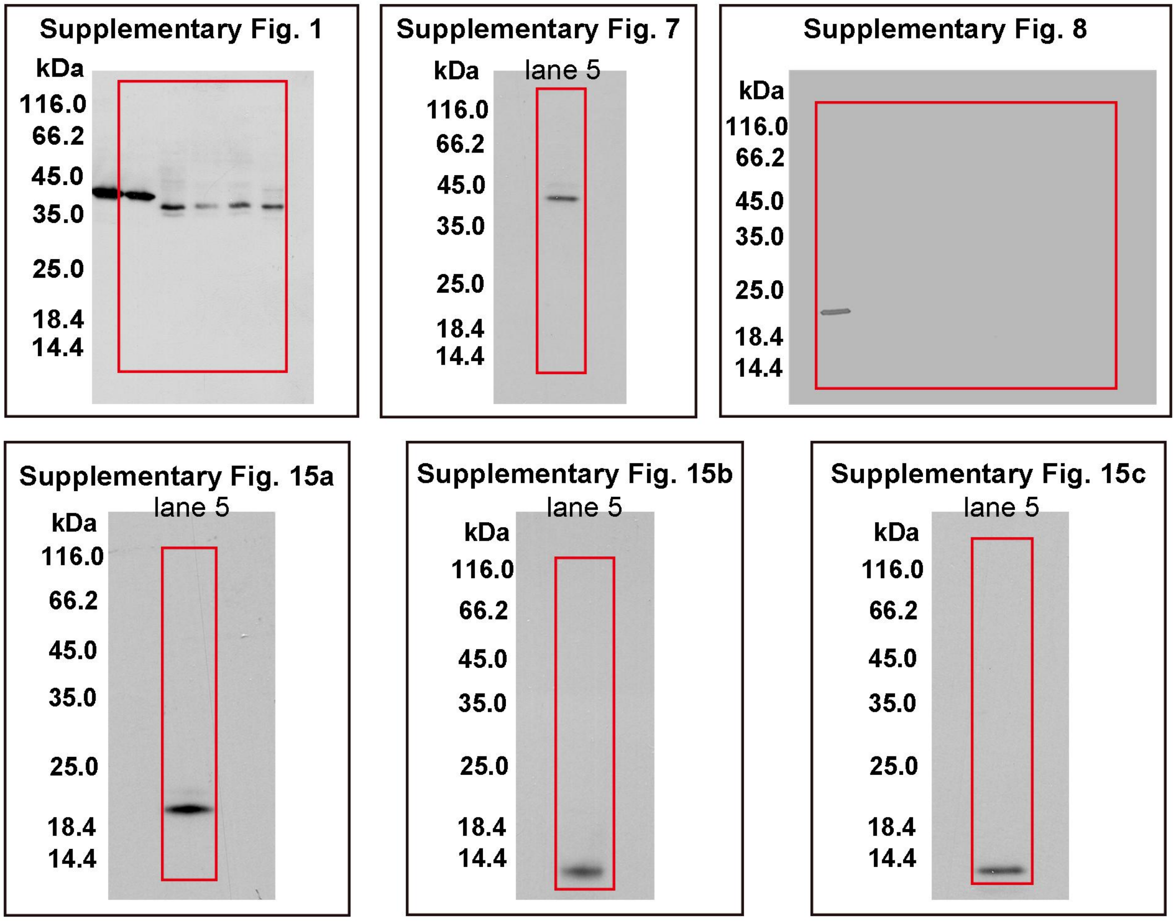

Supplementary Fig. 20 Unprocessed original blot images represented in Supplementary Figs. 1, 7, 8 and 15.

Supplementary Table 1 Nuclear localization signal of human and mouse ELAVL1 and zebrafish ELAVL1a proteins analyzed by PSORTII. Zebrafish ELAVL1a contained no typical nuclear localization signal and the NNCN result predicts that it has 55.5% of reliability in cytoplasmic, and the κ-NN result indicates that it has the reliability of 26.1% distributed in nuclear and 60.9% in cytoplasmic.

species	NUCDISC			NNCN		k-NN	
	pat4	pat7	bipartite	prediction	Reliability	nuclear	cytoplasmic
human	none	none	none	cytoplasmic	70.6	17.40%	69.60%
mouse	none	none	none	cytoplasmic	70.6	17.40%	69.60%
zebrafish	none	none	none	cytoplasmic	55.5	26.10%	60.90%
