# Supplementary material for: ELAVL1a is an immunocompetent protein that protects zebrafish embryos from bacterial infection
Source: Commun Biol. 2021 Feb 26;4:251. doi: 10.1038/s42003-021-01777-z (PMC7910469; doi:10.1038/s42003-021-01777-z)
Supplement: Supplementary file 3 — Description of Additional Supplementary Files [file 42003_2021_1777_MOESM3_ESM.pdf]

Description of Additional Supplementary Files

File name: Supplementary Data 1
Description: Sequences of primers used in this study

File name: Supplementary Data 2
Description: Data used to generate the charts and graphs.
