# Supplementary material for: ELAVL1a is an immunocompetent protein that protects zebrafish embryos from bacterial infection
Source: Commun Biol. 2021 Feb 26;4:251. doi: 10.1038/s42003-021-01777-z (PMC7910469; doi:10.1038/s42003-021-01777-z)
Supplement: Supplementary file 6 — Reporting Summary [file 42003_2021_1777_MOESM6_ESM.pdf]

nature research

Corresponding author(s): Shicui Zhang

Last updated by author(s): Dec 11, 2020

Reporting Summary

Nature Research wishes to improve the reproducibility of the work that we publish. This form provides structure for consistency and transparency in reporting. For further information on Nature Research policies, see our Editorial Policies and the Editorial Policy Checklist.

Statistics

For all statistical analyses, confirm that the following items are present in the figure legend, table legend, main text, or Methods section.

n/a	Confirmed	
☐	☒	The exact sample size (*n*) for each experimental group/condition, given as a discrete number and unit of measurement
☐	☒	A statement on whether measurements were taken from distinct samples or whether the same sample was measured repeatedly
☐	☒	The statistical test(s) used AND whether they are one- or two-sided<br>*Only common tests should be described solely by name; describe more complex techniques in the Methods section.*
☒	☐	A description of all covariates tested
☒	☐	A description of any assumptions or corrections, such as tests of normality and adjustment for multiple comparisons
☐	☒	A full description of the statistical parameters including central tendency (e.g. means) or other basic estimates (e.g. regression coefficient) AND variation (e.g. standard deviation) or associated estimates of uncertainty (e.g. confidence intervals)
☒	☐	For null hypothesis testing, the test statistic (e.g. *F*, *t*, *r*) with confidence intervals, effect sizes, degrees of freedom and *P* value noted<br>*Give P values as exact values whenever suitable.*
☒	☐	For Bayesian analysis, information on the choice of priors and Markov chain Monte Carlo settings
☒	☐	For hierarchical and complex designs, identification of the appropriate level for tests and full reporting of outcomes
☒	☐	Estimates of effect sizes (e.g. Cohen's *d*, Pearson's *r*), indicating how they were calculated

Our web collection on statistics for biologists contains articles on many of the points above.

Software and code

Policy information about availability of computer code

Data collection	No customized software was used. All immunohistochemical staining images were acquired by using Leica Application Suite software; WinMDI, version 2.9, software (Scripps Research Inst., San Diego, CA, USA) was used to collect flow cytometry data; Western blot data were collected by ImageJ (V1.8.0, NIH).
Data analysis	No customized software was used. GraphPad Prism 5 (Graphpad Software, La Jolla, CA, USA) to plot data and perform statistical analysis.

For manuscripts utilizing custom algorithms or software that are central to the research but not yet described in published literature, software must be made available to editors and reviewers. We strongly encourage code deposition in a community repository (e.g. GitHub). See the Nature Research guidelines for submitting code & software for further information.

Data

Policy information about availability of data

All manuscripts must include a data availability statement. This statement should provide the following information, where applicable:

- Accession codes, unique identifiers, or web links for publicly available datasets
- A list of figures that have associated raw data
- A description of any restrictions on data availability

All data and accession codes are available in the main text and the additional files submitted with the manuscript.

Field-specific reporting

Please select the one below that is the best fit for your research. If you are not sure, read the appropriate sections before making your selection.

☒ Life sciences ☐ Behavioural & social sciences ☐ Ecological, evolutionary & environmental sciences

For a reference copy of the document with all sections, see nature.com/documents/nr-reporting-summary-flat.pdf

Life sciences study design

All studies must disclose on these points even when the disclosure is negative.

Sample size	For western blotting analysis, 5 wild-type zebrafish and 30 embryos of wild-type zebrafish at each stage were used. For immunohistochemistry analysis, 10 embryos of wild-type zebrafish at each stage were used. For cloning of elavl1a gene, 60 embryos of wild-type zebrafish were used. For gene expression analysis, 5 wild-type zebrafish and 30 embryos of wild-type zebrafish at each stage were used. For Whole-mount in situ hybridization, 10 embryos of wild-type zebrafish at each stage were used. For assay for antibacterial activity of ELAVL1a in embryos/larvae, 200 embryos of wild-type zebrafish were used.
Data exclusions	No data were excluded from the analyses.
Replication	n this study, three independent experiments were replicated t verify the findings and all the attempts were successful.
Randomization	Samples were selected randomly.
Blinding	The investigator was totally blind for the data sampling.

Reporting for specific materials, systems and methods

We require information from authors about some types of materials, experimental systems and methods used in many studies. Here, indicate whether each material, system or method listed is relevant to your study. If you are not sure if a list item applies to your research, read the appropriate section before selecting a response.

Materials & experimental systems

n/a	Involved in the study	
☐	☒	Antibodies
☒	☐	Eukaryotic cell lines
☒	☐	Palaeontology and archaeology
☐	☒	Animals and other organisms
☒	☐	Human research participants
☒	☐	Clinical data
☒	☐	Dual use research of concern

Methods

n/a	Involved in the study	
☒	☐	ChIP-seq
☐	☒	Flow cytometry
☒	☐	MRI-based neuroimaging

Antibodies

Antibodies used	Mouse anti-human ELAVL1 antibody (ABIN577055; Clonegene, Hartford, Connecticut, USA);<br>Mouse IgG1 isotype control antibody (ABIN457406; Clonegene, Hartford, Connecticut, USA);<br>anti-β-actin Mouse Monoclonal antibody (CW0264M; Cwbio, Beijing, China).
Validation	Mouse anti-human ELAVL1 antibody (ABIN577055; Clonegene, Hartford, Connecticut, USA) have been validated by previous study and further validated in this study; Mouse IgG1 isotype control antibody (ABIN457406; Clonegene, Hartford, Connecticut, USA) and anti-β-actin Mouse Monoclonal antibody (CW0264M; Cwbio, Beijing, China) both have the validation statements on the manufacturer's websites

Animals and other organisms

Policy information about studies involving animals; ARRIVE guidelines recommended for reporting animal research

Laboratory animals	Zebrafish embryos were all used between 0 and 168 hours post fertilization
Wild animals	The wild-type zebrafish purchased from a local fish dealer and maintained in the containers with well-aerated tap water at 27 ± 1 خ .
Field-collected samples	The study did not involve samples collected from the field.
Ethics oversight	All the fish used in the experiments were treated in accordance with the guidelines of the Laboratory Animal Administration Law of

China, with the permit number SD2007695 approved by the Ethics Committee of the Laboratory Animal Administration of Shandong province.

Note that full information on the approval of the study protocol must also be provided in the manuscript.

Flow Cytometry

Plots

Confirm that:

- [x] The axis labels state the marker and fluorochrome used (e.g. CD4-FITC).
- [x] The axis scales are clearly visible. Include numbers along axes only for bottom left plot of group (a 'group' is an analysis of identical markers).
- [x] All plots are contour plots with outliers or pseudocolor plots.
- [x] A numerical value for number of cells or percentage (with statistics) is provided.

Methodology

Sample preparation

Flow cytometry was used to measure the effect of rELAVL1a on the bacterial membranes of the Gram-positive bacteria M. luteus, B. subtilis and S. aureus and the Gram-negative bacteria E. coli, V. anguillarum and A. hydrophila. The Gram-positive bacteria M. luteus, B. subtilis and S. aureus and the Gram-negative bacteria E. coli and V. anguillarum were cultured in LB medium at 37°C to mid-logarithmic phase and collected by centrifugation at 6000 × g at room temperature for 10 min. The Gram-negative bacterium A. hydrophila was also cultured in tryptic soy broth medium at 28°C to mid-logarithmic phase and collected by centrifugation. After washing three times with PBS, the bacterial pellets were suspended in PBS and adjusted to a density of 1 × 10^6 cells/ml, and then rELAVL1a was added to the bacterial suspensions, yielding final concentrations of 10, 20 and 40 μg/ml. For control, the bacteria were mixed with PBS alone. The mixtures were incubated at 28°C for 2 h and fixed with 10 M propidium iodide (PI; a DNA-intercalating agent and a fluorescent molecule; Sigma-Aldrich, St. Louis, MO, USA) solution under dark conditions at 4°C for 15 min.

Instrument

FC500MPL flow cytometer (Beckman, Brea, CA, USA).

Software

WinMDI, version 2.9, software (Scripps Research Inst., San Diego, CA, USA).

Cell population abundance

For analysis, at least 10,000 total events were acquired for all analysis.

Gating strategy

All cells were gated based on forward and side-scatter characteristics to limit debris.

- [x] Tick this box to confirm that a figure exemplifying the gating strategy is provided in the Supplementary Information.
